# Compatibility Evaluation and Anatomical Observation of Melon Grafted Onto Eight *Cucurbitaceae* Species

**DOI:** 10.3389/fpls.2021.762889

**Published:** 2021-10-20

**Authors:** Mu Xiong, Changjin Liu, Liping Guo, Jin Wang, Xiangshuai Wu, Ling Li, Zhilong Bie, Yuan Huang

**Affiliations:** Key Laboratory of Horticultural Plant Biology, Ministry of Education, College of Horticulture and Forestry Sciences, Huazhong Agricultural University, Wuhan, China

**Keywords:** melon, graft compatibility, *Cucurbitaceae*, starch accumulation, vascular development, callose deposition, necrotic layer

## Abstract

Melon (*Cucumis melo*) is one of the top 10 fruits in the world, and its production often suffers due to soil-borne diseases. Grafting is an effective way to solve this problem. However, graft incompatibility between scion and rootstock limits the application of melon grafting. In this study, the melon was grafted onto eight *Cucurbitaceae* species (cucumber, pumpkin, melon, luffa, wax gourd, bottle gourd, bitter gourd, and watermelon), and graft compatibility evaluation and anatomical observation were conducted. Taking melon homo-grafted plants as control, melon grafted onto cucumber and pumpkin rootstocks was compatible, while melon grafted onto luffa, wax gourd, bottle gourd, bitter gourd, and watermelon rootstocks was incompatible based on the scion dry weight on day 42 after grafting. Meanwhile, we found that starch–iodine staining of scion stem base is an index to predict graft compatibility earlier, on day 14 after grafting. Further, microsection observations showed that there was more cell proliferation at graft junction of melon hetero-grafted combinations; vascular reconnection occurred in all graft combinations. However, excess callose deposited at graft junction resulted in the blockage of photosynthate transport, thus, leading to starch accumulation in scion stem base, and finally graft incompatibility. In addition, undegraded necrotic layer fragments were observed at graft junctions of melon grafted onto incompatible bitter gourd and watermelon rootstocks. The above results provide clues for the selection and breeding of compatible *Cucurbitaceae* rootstocks of melon and demonstrate that starch accumulation in scion base and callose deposition at graft junction is associated with melon graft compatibility.

## Introduction

Grafting, as an asexual plant propagation technology, has been applied for about 3000 years (Melnyk and Meyerowitz, 2015). It is widely used in agriculture as it enhances abiotic/biotic stress resistance, extends the harvesting period, improves fruit yield and quality, adjusts flowering time, and improves fruit tree architecture (Lee et al., 2010; Louws et al., 2010; Huang et al., 2011; Nawaz et al., 2016, 2017; Ceballos et al., 2017; Niu et al., 2018; Souza et al., 2018; Zhong et al., 2018; Gautier et al., 2019; Migicovsky et al., 2019). However, graft incompatibility usually occurs between scion and rootstock, leading to a low survival ratio, abnormal growth, and low yield (Ren et al., 2018).

Graft incompatibility has been reported in various horticultural species. When watermelon was grafted onto different pumpkin rootstocks, the survival rate of graft incompatible combinations was significantly reduced (Yetisir and Sari, 2003; Yetisir et al., 2007). Litchi scion leaves turned yellow and the scion base swelled 6 weeks after being grafted onto incompatible rootstocks (Chen Z. et al., 2016, 2017). The survival ratio could reach 87 and 90% on day 30 but reduced to 0 and 7% on day 180 after honey pomelo (*Citrus grandis*) cv. ‘Hongmianmiyou’ and ‘Huangjinmiyou’ were grafted onto *Poncirus trifoliata* (Gong et al., 2016). Tomato/pepper healing junction showed the asynchronous stem bulging 30 days after grafting (Masayuki et al., 2000; Thomas et al., 2021), which was also observed in graft incompatible melon (Cohen et al., 2007; Zhou et al., 2018).

Melon (*Cucumis melo* L.) is an important horticultural crop that belongs to the *Cucurbitaceae* family. Melon often suffers due to soil-borne pathogens. Intraspecific grafting in a melon can be used to avoid damage caused by wilt pathogens, without loss of yield and fruit quality, but cannot suppress root and stem rot diseases (Cohen et al., 2007). Pumpkin rootstocks provide non-specific and efficient protection against those pathogens, and against some abiotic stresses (Cohen et al., 2007; Shang et al., 2016). The differences between melon scion and pumpkin rootstock in water absorption and sugar distribution were not correlated with graft incompatibility on day 14 but on day 24 after grafting (Aloni et al., 2008). The problem of an unbalanced distribution of sugar was also reported by Camalle et al. (2021), who found that there was over-accumulation of sugars and sugar alcohols in the scion of melon grafted onto incompatible pumpkin rootstock (Ki/r53), as compared with compatible pumpkin homo-grafted combination (r53/r53). However, the *Cucurbitaceae* family contains ∼1000 species, which includes many important vegetables and fruits, such as cucumber, melon, wax gourd, watermelon, pumpkin, bitter gourd, luffa, and bottle gourd (Guo et al., 2020). Previous studies on melon graft compatibility were limited to several rootstock species, such as pumpkin and bottle gourd, and a comprehensive evaluation and anatomical observation of compatibility between melon and a range of *Cucurbitaceae* species are lacking.

Successful vascular reconnection is regarded as a landmark event of graft healing (Melnyk, 2017). In amaranth/tomato (Xiang et al., 1992), soybean/pumpkin (Sun et al., 2015), arabidopsis/tomato (Flaishman et al., 2008), and arabidopsis/chrysanthemum (Notaguchi et al., 2020), the persistent necrotic layer of graft junction prevented the vascular bundle differentiation and reconnection, resulting in graft failure. When cucumber was grafted onto incompatible pumpkin rootstocks, its necrotic layer disappeared later than when grafted onto compatible rootstocks (Xu et al., 2016). However, after arabidopsis was grafted onto cabbage, radish, and tobacco, callus was formed at the graft junction, but there were few complete vascular bundles (Flaishman et al., 2008; Notaguchi et al., 2020). The vascular bundles of incompatible grafted combinations in litchi reconnected normally at the early stage, but gaps were formed at the later stage (Chen Z. et al., 2016, 2017). Meanwhile, Thomas et al. (2021) observed parenchymatous callus formation at the junction of compatible homo-grafted tomato and pepper, and incompatible hetero-grafted tomato/pepper, and pepper/tomato; however, the delayed vascular progression and xylem discontinuity occurred in the incompatible hetero-grafted tomato/pepper, and pepper/tomato combinations. Graft incompatibility also affects the xylem and/or the phloem functionality, and hence, the bidirectional transport of photoassimilates, hormones, mineral nutrients, and water is negatively affected (Schoning and Kollmann, 1997; Espen et al., 2005; Kawaguchi et al., 2008; Camalle et al., 2021). The transport of photoassimilates to the roots was blocked on day 78 after grafting in incompatible peach/plum combinations (Espen et al., 2005). In *in vitro* hetero-grafts, the non-transporting sieve-tubes were observed at the junction of *Vicia* grafted onto *Helianthus* (Schoning and Kollmann, 1997). Thus, it is essential to observe the anatomical structure of the graft junction of melon grafted onto different species.

In this study, we evaluated the graft compatibility of melon grafted onto eight *Cucurbitaceae* species and found that starch–iodine staining was a key index, which could be used to predict graft compatibility earlier. Excess callose was deposited in newborn phloem of incompatible combinations, leading to starch accumulations in the scion base. Undegraded necrotic layer fragments were observed at the graft junction of melon grafted onto incompatible bitter gourd and watermelon rootstocks. This study provides useful clues for the selection and breeding of melon rootstock, and more insights to understand the graft compatibility mechanism of melon.

## Materials and Methods

### Plant Material and Grafting

In this study, eight *Cucurbitaceae* species (cucumber, pumpkin, melon, luffa, wax gourd, bottle gourd, bitter gourd, and watermelon) including 40 cultivars were used to evaluate the melon graft compatibility. A Xinjiang local cultivar, ‘Akekekouqi,’ was used as the melon scion. The detailed information on these cultivars is provided in Supplementary Table 1.

The experiments were conducted in 2021 at the National Center of Vegetable Improvement in Huazhong Agricultural University, Central China (30°27′N, 114°20′E, and altitude 22 m above sea level). The seeds were sown in the 50-cell plug trays. Seedlings were cultivated with a day/night (14/10 h) cycle at 28/18°C, and 60–70% relative humidity, in a climate chamber. When the scion cotyledons had fully opened and the first true leaf of rootstocks had fully unfolded, one cotyledon grafting was performed as described by Hassell et al. (2008). The grafted seedlings were then maintained under the conditions described previously (Liu et al., 2021). Briefly, the grafted seedlings were maintained under complete darkness on day 1, low light intensity (80 μmol m^–2^ s^–1^, 14/10 h photoperiod) from day 2 to day 7, and normal light intensity (170 μmol m^–2^ s^–1^, 14/10 h photoperiod) from day 7. The light source was a full-spectrum LED tube light T8 (Gexinlai Optoelectronics Technology Co., Ltd., China). The temperatures were kept at 28/18°C (day/night) during graft healing. The humidity was kept above 95% during the first 5 days, and decreased to 85% from day 6 to day 10, and then to 70% from day 10. For long-term evaluation experiments, grafted seedlings were transplanted into plastic pots on day 14 after grafting, each containing 10 L of the substrate (peat: vermiculite: perlite = 1:1:1, v/v). Each pot contained one grafted seedling. The pots were arranged at 150 cm row spacing, spaced 50 cm apart in a greenhouse, from April to May 2021. The average day/night temperature was 28/18°C. Plants were irrigated with a full-strength Hoagland nutrient solution.

### Determination of Scion Dry Weight, Scion Height, and Scion and Rootstock Stem Diameters

For scion dry weight determination, three plants per replicate were harvested on day 42 after grafting (28 days after transplanting). The scion samples were dried in a forced-air oven at 85°C for 96 h and then weighted. Scion height and scion and rootstock diameters were determined on day 14, day 21, day 28, and day 42 after grafting. Scion and rootstock diameters were determined at 5 mm above or below the graft junction. Stem diameter ratio = (scion diameter/rootstock diameter).

### Determination of Leaf Area and Root Length Ratio, and Leaf SPAD

The 14-day-old grafted plants were analyzed. Leaf area ratio = (true leaf area of melon hetero-grafted plants/true leaf area of melon homo-grafted plants). Root length ratio = (root length of melon hetero-grafted plants/root length of rootstock homo-grafted plants). Leaf area and root length were determined by WinRHIZO (Regent, Canada). SPAD (relative chlorophyll value) was determined using the second true leaf from the top with SPAD-502 Chlorophyll Meter (Konica Minolta, Japan).

### Starch–Iodine Staining of Scion and Rootstock Stem

Plants were sampled on day 14 and day 42 after grafting. To conduct the starch–iodine staining, 2 mm stem above and below the graft junction were cut as the scion and rootstock respectively. The collected samples were placed in 75% ethanol for 24 h to decolorize. After washing the decolorized samples using ddH_2_O_2_ for 1 min, they were placed in I_2_–KI solution (24 mg I_2_, 96 mg KI/ml) for 1 min, followed by washing with ddH_2_O_2_ for 1 min. The stained stem was imaged using a stereoscopic microscope (Olympus SZ61, Olympus, Japan). Starch–iodine staining area ratio = (staining area/transected stem area). The area was analyzed using ImageJ^1^.

### Anatomical Observation of Graft Junction

Graft junctions were collected on day 14 after grafting. The collected samples were placed in 70% FAA (Formaldehyde-acetic acid-ethanol Fixative, Formaldehyde: Acetic acid: Ethanol = 1: 1: 18, volume ratio) for 24 h, and then stored in 70% ethanol at 4°C. The paraffin section was performed as described by El-Gazzar et al. (2017). Samples were sectioned to 8 μm vertically using a rotary microtome (Leica RM2255, Leica, Germany), dewaxed, rehydrated, cleaned, stained with 1% safranin, counterstained with 1% fast green, and then fixed with neutral balata. Sections were imaged using a positive fluorescence microscope (Leica DM6B, Leica, Germany). The vibratome section was performed as described by Wang et al. (2018). Samples were embedded as tissues into 4% agarose and sectioned to 100 μm vertically using an automatic vibratome (Leica VT1200S, Leica, Germany). Lignin was stained for 5 min using 0.01% Basic fuchsin and imaged using a confocal laser scanning microscope (Leica SP8, Leica, Germany). Callose was stained for 1 h using 0.01% aniline blue in 150 mM KH_2_PO_4_ (pH = 9.5) without light and imaged using a confocal laser scanning microscope (Leica SP8, Leica, Germany). For lignin, fluorescence was detected at 552 nm excitation, 580–630 nm emission wavelength; for callose, fluorescence was detected at 405 nm excitation, 505–545 nm emission wavelength.

### Statistical Analysis

All data were analyzed using SPSS 20.0 software (SPSS Inc., Chicago, IL, United States). The significance analysis was performed using Student’s *t*-test (*p* < 0.05). The correlation analysis was done using the Pearson method. The column diagram was made using GraphPad Prism 8.0 (GraphPad Software Inc., San Diego, CA, Canada). The heat map of correlation analysis was made by R.^2^

## Results

### Scion Dry Weight of Melon Grafted Onto Eight *Cucurbitaceae* Species

**FIGURE 1:**
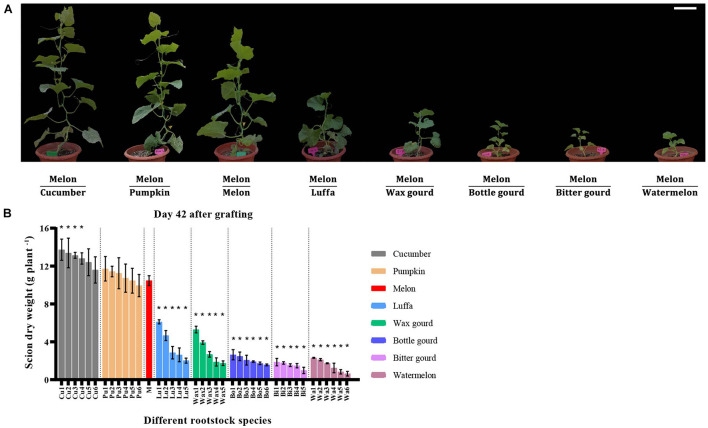
Pictures and scion dry weight of melon grafted onto eight *Cucurbitaceae* species. **(A)** Pictures of melon cv. ‘Akekekouqi’ grafted onto cucumber cv. ‘Jinyou No.35,’ pumpkin cv. ‘Qingyouzhen No.1,’ melon cv. ‘Akekekouqi,’ luffa cv. ‘Sanbi No.6,’ wax gourd cv. ‘Aonong,’ bottle gourd cv. ‘H19,’ bitter gourd cv. ‘Liangku No.1,’ and watermelon cv. ‘Zaojia 8424’ on day 42 after grafting. Scale bar represents 15 cm. **(B)** Scion dry weight of melon cv. ‘Akekekouqi’ grafted onto cucumber (Cu1–Cu6), pumpkin (Pu1–Pu6), melon (M), luffa (Lu1–Lu5), wax gourd (Wax1–Wax5), bottle gourd (Bo1–Bo6), bitter gourd (Bi1–Bi5), and watermelon (Wa1–Wa6) on day 42 after grafting. Asterisks indicate a significant difference between melon homo-grafted plants and hetero-grafted plants on day 42 after grafting using Student’s *t*-test (*p* < 0.05).

Compared with melon homo-grafted plants, hetero-grafted rootstocks affected the scion dry weight (Figure 1). According to scion dry weight, cucumber and pumpkin were graft compatible rootstocks, and for cucumber rootstocks, four out of six cultivars had significantly higher scion dry weight than melon homo-grafted plants and, therefore, had higher compatibility than pumpkin rootstocks (Figure 1B). By contrast, luffa, wax gourd, bottle gourd, bitter gourd, and watermelon rootstocks were graft incompatible with melon, and scion dry weight of all tested cultivars was significantly lower than melon homo-grafted plants (Figure 1B).

### Scion Height and Scion and Rootstock Stem Diameters of Melon Grafted Onto Eight *Cucurbitaceae* Species

**FIGURE 2:**
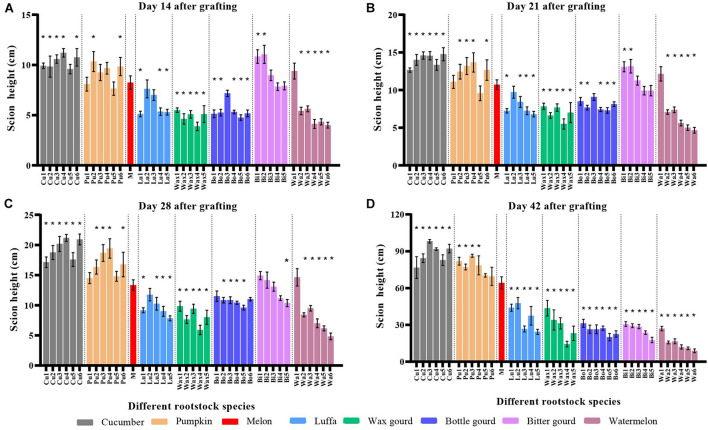
Scion height of melon grafted onto eight *Cucurbitaceae* species. Scion height of melon cv. ‘Akekekouqi’ grafted onto cucumber (Cu1–Cu6), pumpkin (Pu1–Pu6), melon (M), luffa (Lu1–Lu5), wax gourd (Wax1–Wax5), bottle gourd (Bo1–Bo6), bitter gourd (Bi1–Bi5), and watermelon (Wa1–Wa6) on day 14 **(A)**, day 21 **(B)**, day 28 **(C)**, and day 42 **(D)** after grafting. Asterisks indicate a significant difference between melon homo-grafted plants and hetero-grated plants using the Student’s *t*-test (*p* < 0.05).

Melon grafted onto cucumber rootstocks had significantly higher scion height than melon homo-grafted plants on day 14, day 21, day 28, and day 42 after grafting, and only Cu5 rootstock showed no significant difference on day 14 (Figure 2). There was a significant difference in scion height of melon grafted onto different pumpkin rootstocks, but no hetero-grafted combination showed a significantly lower height as compared with melon homo-grafted plants (Figure 2). Luffa, wax gourd, bottle gourd, and watermelon rootstocks showed significance on day 14, day 21, and day 28, but no hetero-grafted combination showed a significantly higher scion height as compared with melon homo-grafted plants (Figures 2A–C); and the scion height of all cultivars of these rootstocks was significantly lower on day 42 (Figure 2D). Bitter gourd 1 and Bitter gourd 2 rootstocks showed significantly higher scion height than melon homo-grafted plants on day 14 and day 21; however, all cultivars showed significantly lower height on day 42 (Figure 2).

**FIGURE 3:**
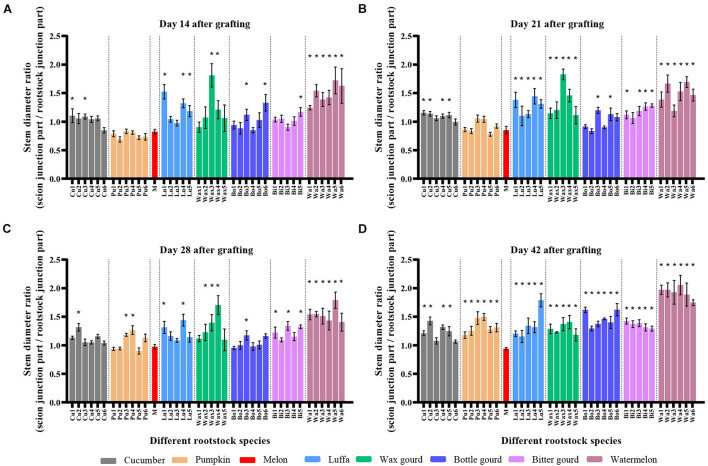
The stem diameter ratio of scion and rootstock of melon grafted onto eight *Cucurbitaceae* rootstocks. The stem diameter ratio of scion and rootstock (above and below 5 mm of the graft junction) of melon cv. ‘Akekekouqi’ grafted onto cucumber (Cu1–Cu6), pumpkin (Pu1–Pu6), melon (M), luffa (Lu1–Lu5), wax gourd (Wax1–Wax5), bottle gourd (Bo1–Bo6), bitter gourd (Bi1–Bi5), and watermelon (Wa1–Wa6) on day 14 **(A)**, day 21 **(B)**, day 28 **(C)**, and day 42 **(D)** after grafting. Asterisks indicate a significant difference between melon homo-grafted plants and hetero-grated plants using the Student’s *t*-test (*p* < 0.05).

The stem diameter of graft junction was usually related to graft compatibility. On day 14 after grafting, the stem diameter ratio showed that five out of six cucumbers, four out of five luffas, four out of five wax gourds, three out of six bottle gourds, four out of five bitter gourd cultivars, and all watermelon rootstocks grafted combinations had thicker scion base than rootstock (Figure 3A). All rootstock species, excluding melon homo-grafted plants, showed a thicker scion base than rootstock on day 42; the watermelon rootstocks grafted combinations had the highest stem diameter ratio of scion/rootstock (Figure 3D). Furthermore, rootstock diameters of incompatible combinations were generally lower than compatible combinations on day 42 (Figures 3A–D; Supplementary Figures 2, 3).

### Leaf Area and Root Length Ratio, and Leaf SPAD of Melon Grafted Onto Eight *Cucurbitaceae* Species

**FIGURE 4:**
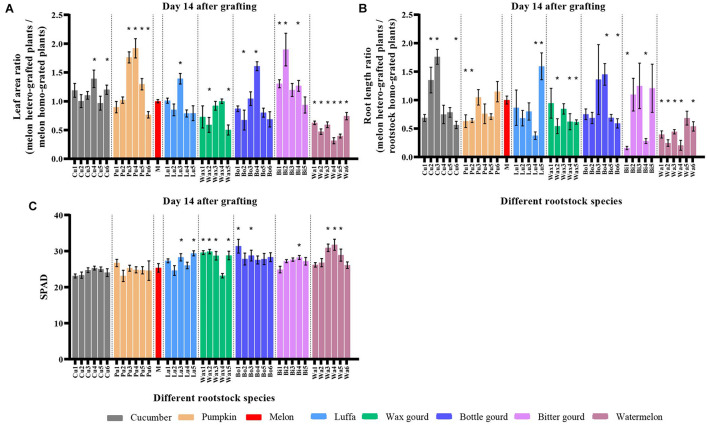
Leaf area, root length, and SPAD of melon grafted onto eight *Cucurbitaceae* rootstocks on day 14 after grafting. Leaf area ratio (**A**, melon hetero-grafted plants/melon homo-grafted plants), root length ratio (**B**, melon hetero-grafted plants/rootstock homo-grafted plants), and SPAD (**C**, relative chlorophyll value) of melon cv. ‘Akekekouqi’ grafted onto cucumber (Cu1–Cu6), pumpkin (Pu1–Pu6), melon (M), luffa (Lu1–Lu5), wax gourd (Wax1–Wax5), bottle gourd (Bo1–Bo6), bitter gourd (Bi1–Bi5), and watermelon (Wa1–Wa6). Asterisks indicate a significant difference between melon or rootstock homo-grafted plants and hetero-grafted plants using Student’s *t*-test (*p* < 0.05).

Compared with melon homo-grafted plants, hetero-grafted rootstocks had different effects on leaf area ratio, where only six cultivars of watermelon rootstocks showed a significantly lower ratio on day 14 after grafting (Figure 4A). Compared with rootstock homo-grafted plants, 15 cultivars of 39 hetero-grafted combinations, including one cucumber, two pumpkins, one luffa, three wax gourds, one bottle gourd, two bitter gourds, and five watermelons, had significant negative effects, and only four cultivars including two cucumbers, one luffa, and one bottle gourd had significantly positive effects on root length ratio on day 14 (Figure 4B). For SPAD, there was no significant difference in graft compatible combinations (cucumber and pumpkin), while 12 of 27 graft incompatible combinations (luffa, wax gourd, bottle gourd, bitter gourd, and watermelon) had higher values when compared with melon homo-grafted plants (Figure 4C).

### Starch Accumulation Above and Below the Graft Junction of Melon Grafted Onto Eight *Cucurbitaceae* Species

**FIGURE 5:**
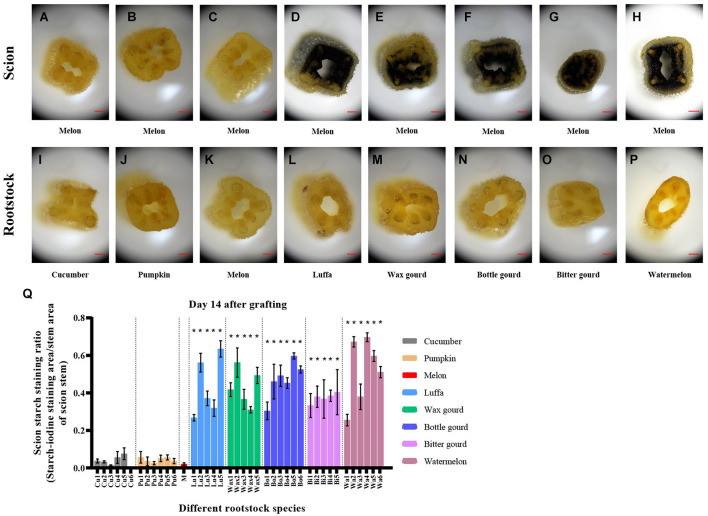
Starch accumulations above and below the graft junction of melon grafted onto eight *Cucurbitaceae* rootstocks on day 14 after grafting. **(A–H)** Starch–iodine staining of scion stem above 2 mm of the graft junction of melon cv. ‘Akekekouqi’ grafted onto cucumber cv. ‘Jinyou No.35,’ pumpkin cv. ‘Qingyouzhen No.1,’ melon cv. ‘Akekekouqi,’ luffa cv. ‘Sanbi No.6,’ wax gourd cv. ‘Aonong,’ bottle gourd cv. ‘H19,’ bitter gourd cv. ‘Liangku No.1,’ and watermelon cv. ‘Zaojia 8424.’ Scale bar represents 500 μm. **(I–P)** Starch–iodine staining of rootstock stem below 2 mm of the graft junction of melon cv. ‘Akekekouqi’ grafted onto cucumber cv. ‘Jinyou No.35,’ pumpkin cv. ‘Qingyouzhen No.1,’ melon cv. ‘Akekekouqi,’ luffa cv. ‘Sanbi No.6,’ wax gourd cv. ‘Aonong,’ bottle gourd cv. ‘H19,’ bitter gourd cv. ‘Liangku No.1,’ and watermelon cv. ‘Zaojia 8424.’ Scale bar represents 500 μm. **(Q)** Scion starch staining ratio (starch–iodine staining area/transected area of scion stem) of melon cv. ‘Akekekouqi’ grafted onto cucumber (Cu1–Cu6), pumpkin (Pu1–Pu6), melon (M), luffa (Lu1–Lu5), wax gourd (Wax1–Wax5), bottle gourd (Bo1–Bo6), bitter gourd (Bi1–Bi5), and watermelon (Wa1–Wa6) on day 14 after grafting. Asterisks indicate a significant difference between melon homo-grafted plants and hetero-grafted plants using the Student’s *t*-test (*p* < 0.05).

Starch–iodine staining can indicate the starch accumulation in plant tissue (Santacruz et al., 2005). According to the staining area, only around 10% area of melon scion stems was stained above 2 mm of the graft junction when grafted onto cucumber, pumpkin, and melon rootstocks on day 14 after grafting, while 27–70% area was stained when the melon was grafted onto luffa, wax gourd, bottle gourd, bitter gourd, and watermelon rootstocks (Figure 5). Only around 10% area of the rootstock stem was stained below 2 mm of the graft junction in all grafted plants on day 14 (Supplementary Figure 4). On day 42 after grafting, the staining tendency was similar as on day 14 after grafting (Supplementary Figure 5).

### Correlation Analysis Between Measured Parameters of Melon Grafted Onto Eight *Cucurbitaceae* Species

**FIGURE 6:**
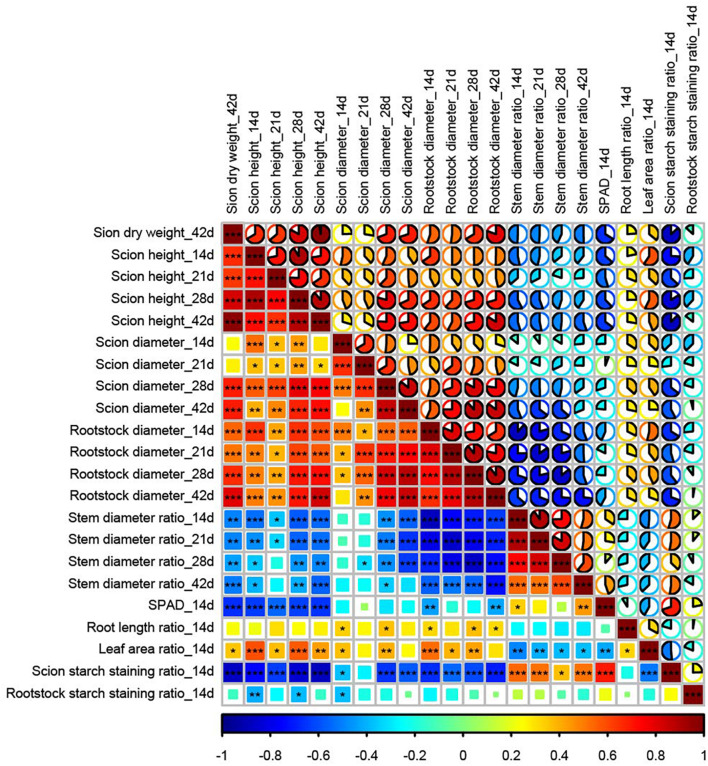
Correlation analysis between parameters in melon grafted onto eight *Cucurbitaceae* rootstocks. Stem diameter ratio: stem diameter above 5 mm of the graft junction/stem diameter below 5 mm of the graft junction. Root length ratio: root length of melon hetero-grafted plants/root length of their rootstock homo-grafted plants. Leaf area ratio: scion leaf area of melon hetero-grafted plants/melon homo-grafted plants. Scion starch staining ratio: starch–iodine staining area/area of transected scion stem above 2 mm of the graft junction. Rootstock starch staining ratio: starch–iodine staining area/area of transected rootstock stem below 2 mm of the graft junction. Different colors, square size, and pie size indicate Pearson’s correlation coefficient. Asterisks indicate significant correlation between two parameters (*p < 0.05, **p < 0.01, ***p < 0.001).

Scion dry weight on day 42 after grafting was significantly correlated with plant height on day 42 (*r* = 0.98^∗∗∗^) and day 28 (*r* = 0.83^∗∗∗^), rootstock diameter on day 42 (*r* = 0.80^∗∗∗^), and scion starch staining ratio on day 14 (*r* = −0.87^∗∗∗^). Scion starch staining ratio was significantly correlated with plant height on day 28 (*r* = −0.84^∗∗∗^) and day 42 (*r* = −0.88^∗∗∗^) (Figure 6). However, other parameters on day 14, including scion height (*r* = 0.66^∗∗∗^), rootstock diameter (*r* = 0.55^∗∗∗^), stem diameter ratio (*r* = −0.49^∗∗^), leaf area ratio (*r* = 0.36^∗^), and SPAD (*r* = −0.66^∗∗∗^) were significantly correlated with scion dry weight on day 42 after grafting (Figure 6).

### Anatomical Observation of the Grafted Junction

**FIGURE 7:**
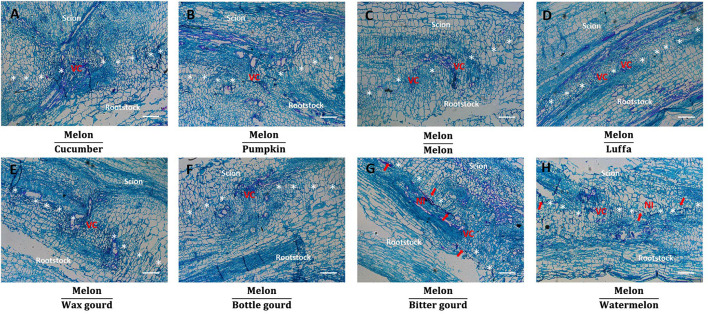
Necrotic layer and vascular reconnection of melon grafted onto eight *Cucurbitaceae* species. **(A–H)** Longitudinal sections of the graft junction of melon cv. ‘Akekekouqi’ grafted onto cucumber cv. ‘Jinyou No.35,’ pumpkin cv. ‘Qingyouzhen No.1,’ melon cv. ‘Akekekouqi,’ luffa cv. ‘Sanbi No.6,’ wax gourd cv. ‘Aonong,’ bottle gourd cv. ‘H19,’ bitter gourd cv. ‘Liangku No.1,’ and watermelon cv. ‘Zaojia 8424’ on day 14 after grafting. Asterisks indicate graft junction. Red arrows indicate undegraded necrotic layer fragments. VC, vascular reconnection; NI, necrotic layer. Scale bar represents 250 μm.

**FIGURE 8:**
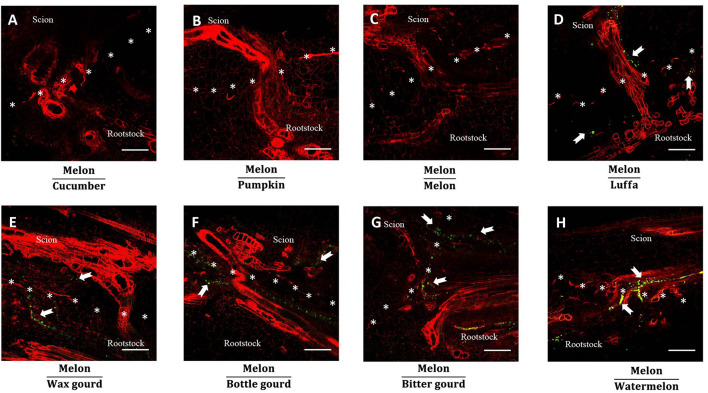
Callose deposition of melon grafted onto eight *Cucurbitaceae* species. **(A–H)** Microscopic observation of the new vascular tissue at graft junction of melon cv. ‘Akekekouqi’ grafted onto cucumber cv. ‘Jinyou No.35,’ pumpkin cv. ‘Qingyouzhen No.1,’ melon cv. ‘Akekekouqi,’ luffa cv. ‘Sanbi No.6,’ wax gourd cv. ‘Aonong,’ bottle gourd cv. ‘H19,’ bitter gourd cv. ‘Liangku No.1,’ and watermelon cv. ‘Zaojia 8424’ on day 14 after grafting. Asterisks indicate graft junction. White arrows indicate callose deposition. Scale bar represents 200 μm.

To observe the graft junction, one cultivar of each rootstock species was selected, using melon as the scion, and their anatomical analyses were performed on day 14 after grafting (Figures 7, 8). We observed the completed newborn vascular bundle passing through the graft junction in all grafted combinations (Figures 7, 8). However, more cell proliferation was observed at graft junctions in hetero-grafted plants when compared to homo-grafted plants. The compatible rootstocks cucumber cv. ‘Jinyou No.35’ and pumpkin cv. ‘Qingyouzhen No.1’ grafted combinations showed more cell proliferation than the incompatible rootstocks luffa cv. ‘Sanbi No.6,’ wax gourd cv. ‘Aonong,’ bottle gourd cv. ‘H19,’ bitter gourd cv. ‘Liangku No.1,’ and watermelon cv. ‘Zaojia 8424’ grafted combinations (Figure 7). We observed the undegraded necrotic layer fragments at the graft junction of bitter gourd and watermelon rootstocks, especially bitter gourd (Figures 7F,G). The aniline blue staining results indicated that excess callose was deposited in new-vascular tissue in luffa, wax gourd, bottle gourd, bitter gourd, and watermelon rootstocks grafted combinations (Figure 8).

## Discussion

### Melon Graft Compatibility in *Cucurbitaceae*

Previous studies on the evaluation of melon graft compatibility mainly focused on a small number of rootstock species, such as wild watermelon, pumpkin, and bottle gourd (Cohen et al., 2007; Aloni et al., 2008; Shang et al., 2016; Zhou et al., 2018; Camalle et al., 2021). In this study, 40 cultivars were selected to evaluate the melon graft compatibility, including eight species of *Cucurbitaceae* (cucumber, pumpkin, melon, luffa, wax gourd, bottle gourd, bitter gourd, and watermelon), which gave a more comprehensive evaluation on graft compatibilities between melon and a range of *Cucurbitaceae* species. Based on the melon scion dry weight on day 42 after grafting, it was found that cucumber and pumpkin were the graft compatible rootstocks with melon, while luffa, wax gourd, bottle gourd, bitter gourd, and watermelon were the graft incompatible rootstocks. Other studies showed that there was a difference in the compatibility of melon grafted onto different pumpkin cultivars (Aloni et al., 2008; Camalle et al., 2021). However, melon cv. ‘Akekekouqi’ was compatible when grafted onto all six pumpkin cultivars in this study; different scion and rootstock combinations could explain the discrepancy. Melon scion growth was normal when compatible rootstocks were used. However, the evaluation of incompatible rootstock combinations should be carefully done. For example, bitter gourd rootstock combinations showed higher scion height on day 14 and day 21, while on day 42, the scion height was significantly lower than that of the compatible rootstocks (Figure 2). Therefore, the graft compatibility evaluation of melon should be done over a relatively long time period if only considering plant growth parameters.

### Starch–Iodine Staining Is an Index That Can Be Used to Evaluate the Melon Graft Compatibility Earlier

Graft incompatibility results in an inhibition of plant growth and physiological function, such as plant height suspension, swollen graft junction, and lower photosynthesis (Cohen et al., 2007; Kawaguchi et al., 2008; Xu et al., 2015; Chen Z. et al., 2016). According to our study, the scion height between incompatible and compatible combinations cannot be accurately distinguished until day 42 after grafting for all species. Rootstock stem diameter showed a decreased trend between compatible and incompatible combinations on day 42. Although the correlation heat map showed that plant height on day 42 and rootstock diameter on day 42 were significantly correlated with scion dry weight, they cannot be used as an index to evaluate the melon graft compatibility earlier than scion dry weight.

For the early physiological response on day 14 after grafting, we could not see lower values of SPAD (relative chlorophyll value) in all incompatible graft combinations, which was inconsistent with the values of Xu et al. (2015), who reported the decrease in chlorophyll fluorescence levels and chlorophyll contents on day 25 after grafting in incompatible cucumber graft combinations. Chen Z. et al. (2016) reported the decrease in net photosynthetic rate and chlorophyll contents after 6 months of litchi grafted onto incompatible rootstocks. We thought that in this study, more time was needed to observe the decrease in SPAD because the decrease in photosynthesis of incompatible graft cucumber was noticed on day 25 after grafting (Xu et al., 2015). In addition, other candidate indexes on day 14, such as leaf area ratio, root length ratio, scion diameter, scion height, rootstock diameter, stem diameter ratio, and rootstock starch staining ratio, were not strongly correlated with scion day weight on day 42 after grafting; the scion dry weight is a key parameter to evaluate melon graft compatibility. Therefore, the above indexes are not suitable to evaluate the melon graft compatibility at an earlier stage.

Sugar response ranged from asymmetric to symmetric was the main event in graft healing development (Melnyk et al., 2018). A previous study showed that there was an accumulation of sugars and sugar alcohols, such as glucose, fructose, galactose, and sucrose, in the scion of melon grafted onto incompatible rootstocks (Camalle et al., 2021). Meanwhile, when plants experience huge fluctuations in available carbon, such as that caused by a photosynthetic rate change, abiotic stress, or other unusual situations, they will accumulate and remobilize starch to slow down changes in the carbon balance (Gibon et al., 2009; Stitt et al., 2010). For example, increased sugar levels in leaves can activate the AGPase activity and promote the synthesis of starch during the day (Geigenberger et al., 2005). Starch, as a storage carbohydrate deposited in plant tissues, can be characterized using Iodine solution (Santacruz et al., 2005). Therefore, we tried to build the starch–iodine staining to evaluate the graft compatibility. Based on starch–iodine staining results, the starch accumulation was asymmetric between scion and rootstock in incompatible combinations, while symmetric starch accumulation in compatible combinations was observed on day 14 after grafting. Scion stem starch–iodine staining on day 14 was significantly correlated with scion dry weight on day 42 after grafting (Figure 6), suggesting that it was an index to evaluate the melon compatibility earlier, instead of long-time measurement of scion dry weight, height, and rootstock stem diameter. Therefore, in the future, it would be convenient and efficient to evaluate the graft compatibility of melon earlier (on day 14 after grafting) by a starch–iodine staining of scion stem base, and since this procedure can be done at the seedling stage, even when the plants are still in the plug tray, there is no doubt that the cultivation space, resources, labor input, and evaluation time will be saved.

Compared to plant growth, plant metabolism responds earlier to the internal changes and environmental stimulus (Chen L. et al., 2016; Chen et al., 2017; Zhang et al., 2019). In *de novo* root regeneration of *Arabidopsis*, jasmonic acid (JA) and indole-3-acetic acid (IAA) accumulated within 4 h, but new roots appeared at least 6 days later (Chen L. et al., 2016; Zhang et al., 2019). Litchi showed a significant difference in IAA content between graft compatible and incompatible combinations at 2 h after grafting, but a difference in plant growth was observed for more than 30 days (Chen et al., 2017). Compared to graft compatible combinations, incompatible combinations accumulated excess starch at graft junction, but not all showed significant lower value in plant height and SPAD on day 14 after grafting (Figures 2, 4, 5), indicating that graft incompatibility induced significant differences in starch metabolism, which emerged earlier than in plant growth. In incompatible combinations of luffa, wax gourd, bottle gourd, and watermelon, there were significant differences in scion starch staining ratio between some cultivars in individual species on day 14 after grafting (Figure 5), such as Lu2 and Lu5 compared to Lu1, which could be due to differences in metabolic levels among different cultivars, and this led to varied starch accumulation at an early stage of graft formation in incompatible combinations. Similar results were also observed in melon plants grafted onto different watermelon cultivars, such as higher starch staining ratio but similar plant height in Wa2 and Wa4 compared to Wa3. In conclusion, the difference in metabolic levels of plants due to incompatibility precedes the difference in plant growth, which facilitated us to predict graft incompatibility by metabolic differences earlier.

### Photoassimilate Transport Blockage and Undegraded Necrotic Layer Are Associated With Melon Incompatibility

The graft healing development is accompanied by cell proliferation, cell differentiation, and vascular reconnection at the graft junction (Melnyk et al., 2015). On day 14 after grafting, vascular reconnection events were observed in all grafted combinations. Meanwhile, more cell proliferation at graft junction was observed in hetero-grafted combinations, indicating that scion–rootstock combinations of different species could stimulate callus activities (Figure 7). Compatible combinations showed more cell proliferation than incompatible combinations in hetero-grafted plants, and undegraded necrotic layer fragments were observed at the graft junction of incompatible bitter gourd and watermelon rootstock combinations (Figures 7F,G). Therefore, although the vascular reconnection happened in incompatible combinations, the early graft healing process was still influenced by melon incompatibility, showing reduced callus activities and undegraded necrotic layer fragments at the graft junction, especially in incompatible bitter gourd and watermelon rootstock combinations (Figure 7).

As the photoassimilates, carbohydrates are synthesized from the leaves, transported to the whole body through the phloem sieve, and unloaded at the parenchyma cells through the symplast pathway in plants (Ma et al., 2019; Lu et al., 2020). As mentioned above, excess starch accumulated in the scion base of incompatible combinations, which indicated that there was a blockage of the transport of photoassimilates at the graft junction. These results are consistent with those findings obtained by Schoning and Kollmann (1997) and Espen et al. (2005), who used radiocarbon translocation and 5/6-carboxyfluorescein (CF) experiments to show the transport of photoassimilates *via* the phloem in incompatible grafted combinations. However, these studies only proposed the hypothesis of blockage based on transportation of photoassimilates and did not give detailed information about the blockage of phloem. In this study, microsection observation of fluorescent staining showed that excess callose was deposited in the newborn phloem of incompatible combinations, while no callose deposition was observed in compatible combinations. Callose is mainly distributed in the phloem sieve and participates in regulating the organic transport of plants under unfavorable environments (Wu et al., 2018). Therefore, considering the inhibited plant growth of graft incompatible combinations, we think that callose deposition in new-born phloem, leading to the blockage of transport of photoassimilates, is the main reason for melon graft incompatibility. Further studies are needed to clarify the underlying mechanism of callose deposition in incompatible graft melons.

## Conclusion

In this study, the compatibility of melon grafted onto eight *Cucurbitaceae* species including 40 cultivars was evaluated. Cucumber and pumpkin are graft compatible with melon, while luffa, wax gourd, bottle gourd, bitter gourd, and watermelon are graft incompatible with melon. Callose deposition and undegraded necrotic layer fragments at graft junction were the main reason for melon graft incompatibility. In this study, we proved that graft compatibility can be evaluated earlier (on day 14 after grafting) by a starch–iodine staining. This study provides clues for compatible melon rootstock selection and breeding.

## Data Availability Statement

The original contributions presented in the study are included in the article/Supplementary Material, further inquiries can be directed to the corresponding author.

## Author Contributions

MX, YH, and ZB devised the project. MX, LG, CL, JW, XW, and LL performed the experimental analyses. MX, CL, and YH performed the data analyses. MX and YH wrote the manuscript. All authors approved the final manuscript.

## Conflict of Interest

The authors declare that the research was conducted in the absence of any commercial or financial relationships that could be construed as a potential conflict of interest.

## Publisher’s Note

All claims expressed in this article are solely those of the authors and do not necessarily represent those of their affiliated organizations, or those of the publisher, the editors and the reviewers. Any product that may be evaluated in this article, or claim that may be made by its manufacturer, is not guaranteed or endorsed by the publisher.

## Footnotes

1
https://imagej.nih.gov/ij/

2
https://www.r-project.org/
