# Supplementary material for: Compatibility Evaluation and Anatomical Observation of Melon Grafted Onto Eight Cucurbitaceae Species
Source: Front Plant Sci. 2021 Oct 20;12:762889. doi: 10.3389/fpls.2021.762889 (PMC8563831; doi:10.3389/fpls.2021.762889)
Supplement: Supplementary file 1 [file Data_Sheet_1.PDF]

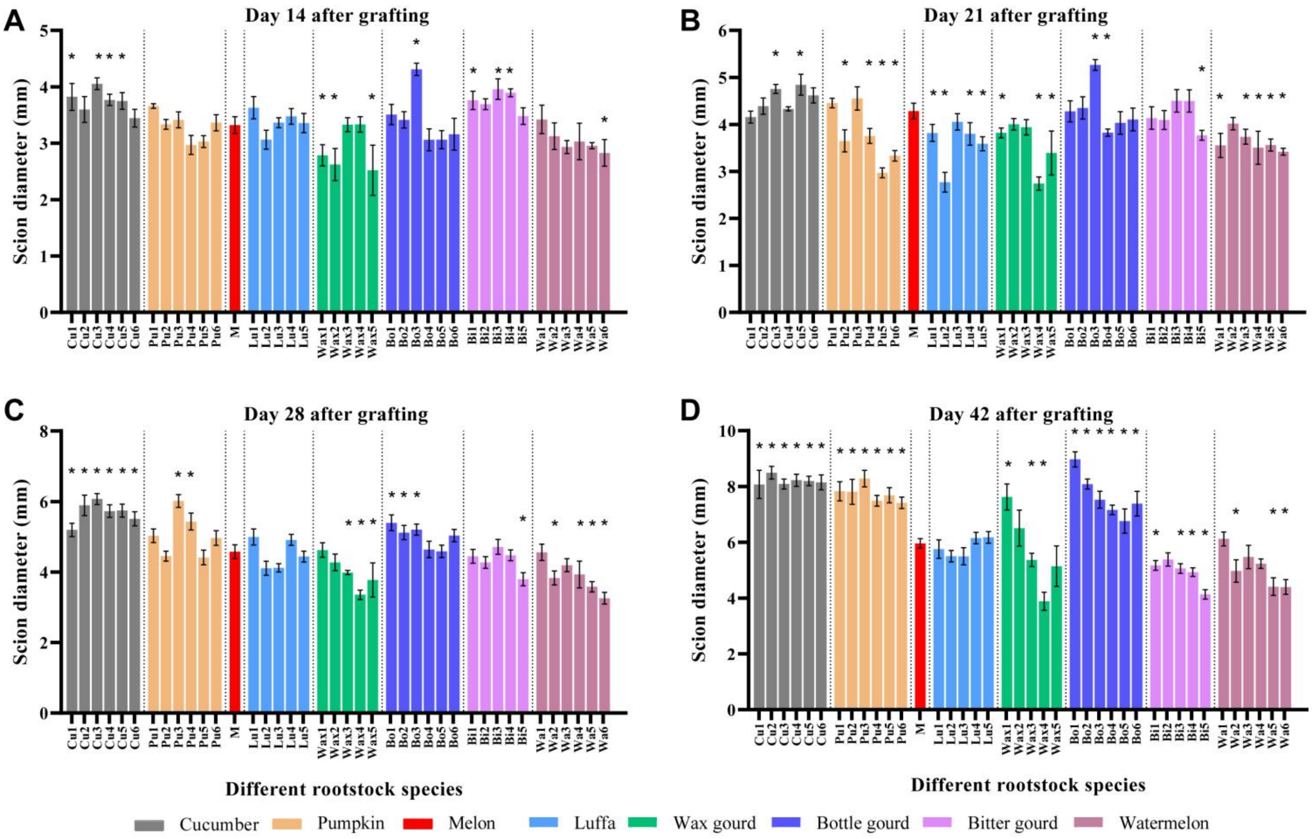

Figure S1. Scion stem diameter of melon grafted onto 8 *Cucurbitaceae* species. Scion stem diameter of melon cv. 'Akekekouqi' grafted onto cucumber (Cu1-Cu6), pumpkin (Pu1-Pu6), melon (M), luffa (Lu1-Lu5), wax gourd (Wax1-Wax5), bottle gourd (Bo1-Bo6), bitter gourd (Bi1-Bi5), and watermelon (Wa1-Wa6) at day 14 **(A)**, day 21 **(B)**, day 28 **(C)**, and day 42 **(D)** after grafting. Asterisks indicate significant difference between melon homo-grafted plants and hetero-grafted plants by Student's t-test ($p < 0.05$).

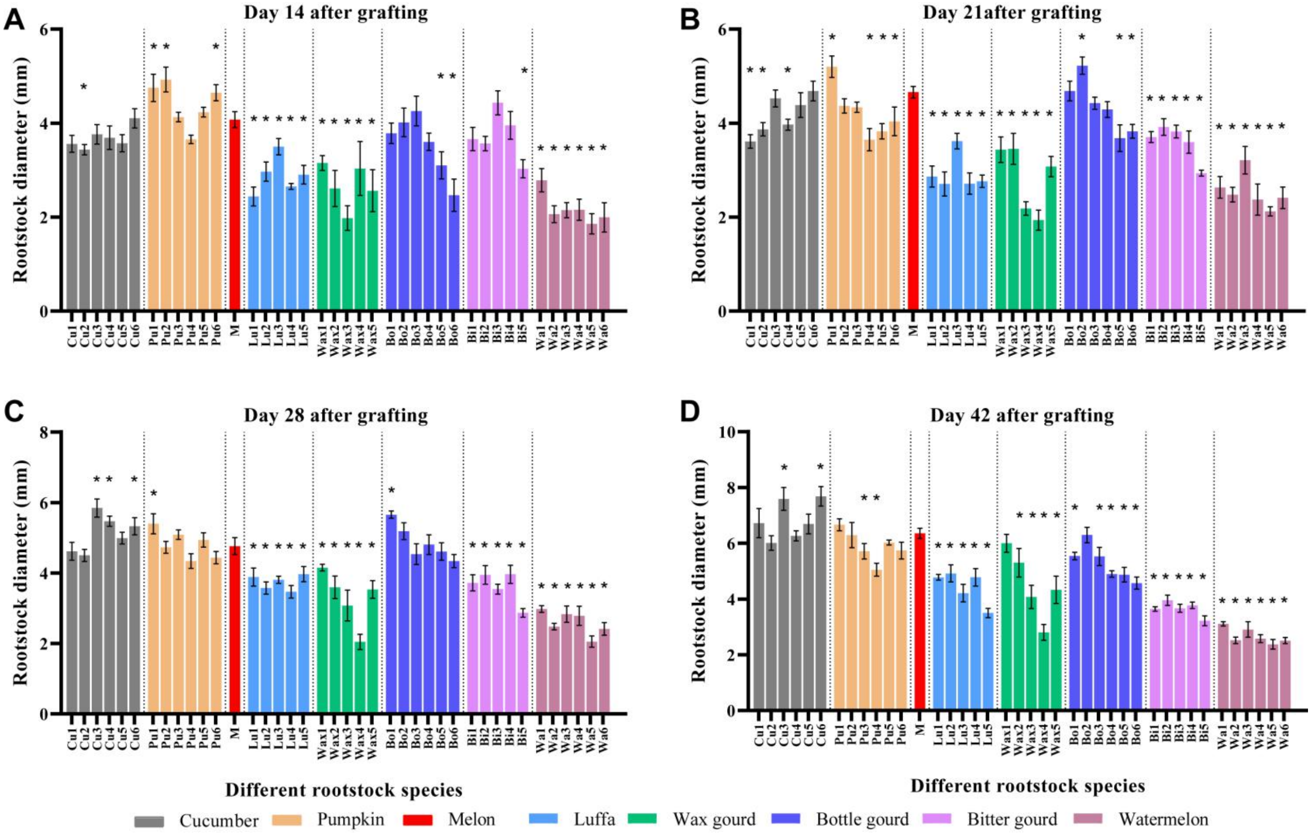

Figure S2. Rootstock stem diameter of melon grafted onto 8 *Cucurbitaceae* rootstocks species. Rootstock stem diameter of melon cv. 'Akekekouqi' grafted onto cucumber (Cu1-Cu6), pumpkin (Pu1-Pu6), melon (M), luffa (Lu1-Lu5), wax gourd (Wax1-Wax5), bottle gourd (Bo1-Bo6), bitter gourd (Bi1-Bi5), and watermelon (Wa1-Wa6) at day 14 **(A)**, day 21 **(B)**, day 28 **(C)**, and day 42 **(D)** after grafting. Asterisks indicate significant difference between melon homo-grafted plants and hetero-grated plants by Student's t-test ($p < 0.05$).

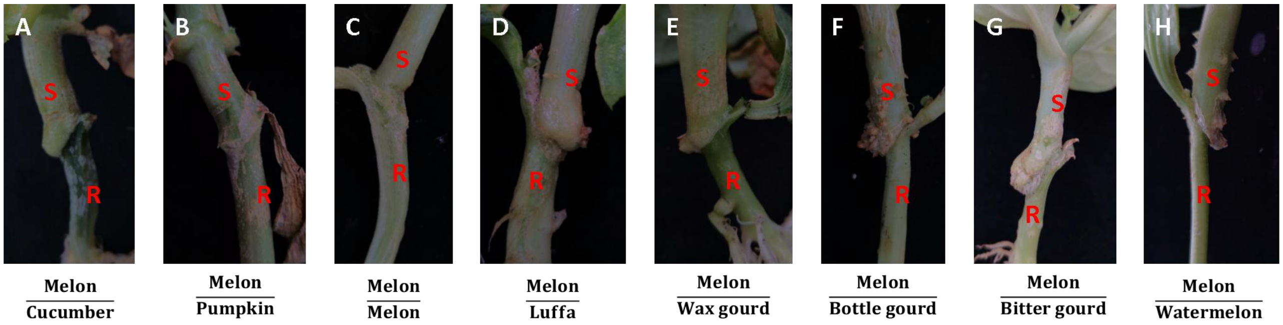

Figure S3. Pictures of graft junction of melon grafted onto 8 *Cucurbitaceae* species at day 42 after grafting. (A-H) Pictures of graft junction of melon cv. ‘Akekekouqi’ grafted onto cucumber cv. ‘Jinyou No.35’, pumpkin cv. ‘Qingyouzhen No.1’, melon cv. ‘Akekekouqi’, luffa cv. ‘Sanbi No.6’, wax gourd cv. ‘Aonong’, bottle gourd cv. ‘H19’, bitter gourd cv. ‘Liangku No.1’, and watermelon cv. ‘Zaojia 8424’.

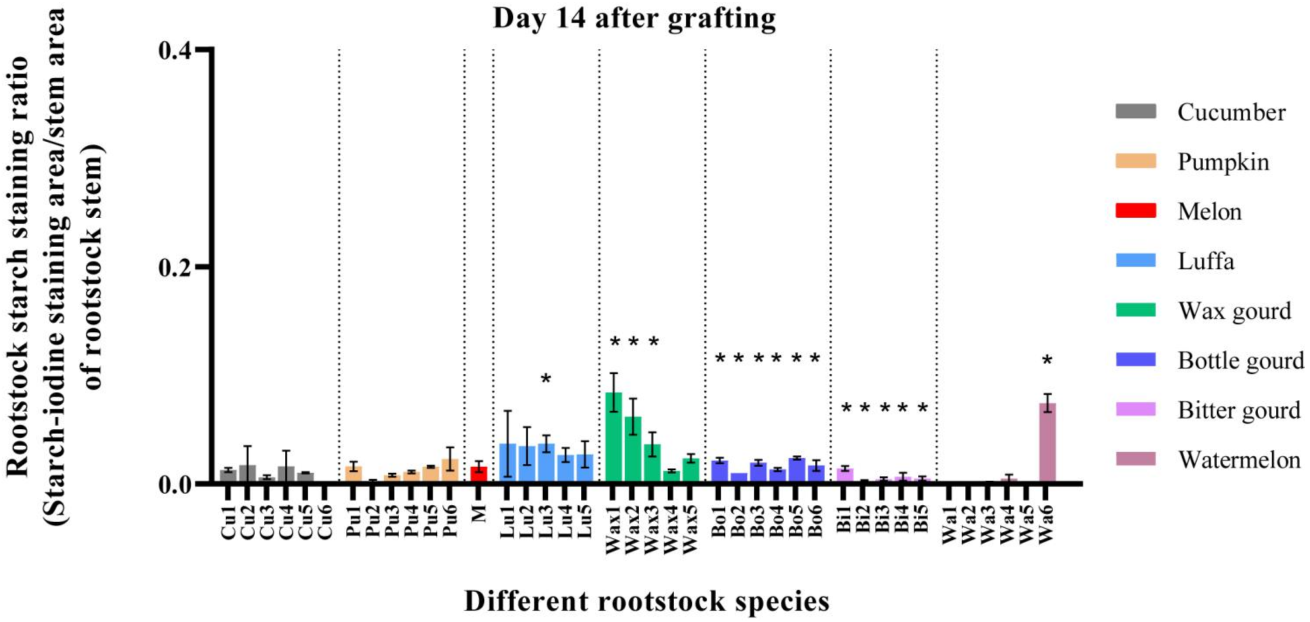

Figure S4. Rootstock starch staining ratio of melon grafted onto 8 *Cucurbitaceae* species at day 14 after grafting. Rootstock starch staining ratio (starch-iodine staining area/stem area of transected rootstock stem) of melon cv. 'Akekekouqi' grafted onto cucumber (Cu1-Cu6), pumpkin (Pu1-Pu6), melon (M), luffa (Lu1-Lu5), wax gourd (Wax1-Wax5), bottle gourd (Bo1-Bo6), bitter gourd (Bi1-Bi5), and watermelon (Wa1-Wa6) at day 14 after grafting. Asterisks indicate significant difference between melon homo-grafted plants and hetero-grated plants by Student's t-test ($p < 0.05$).

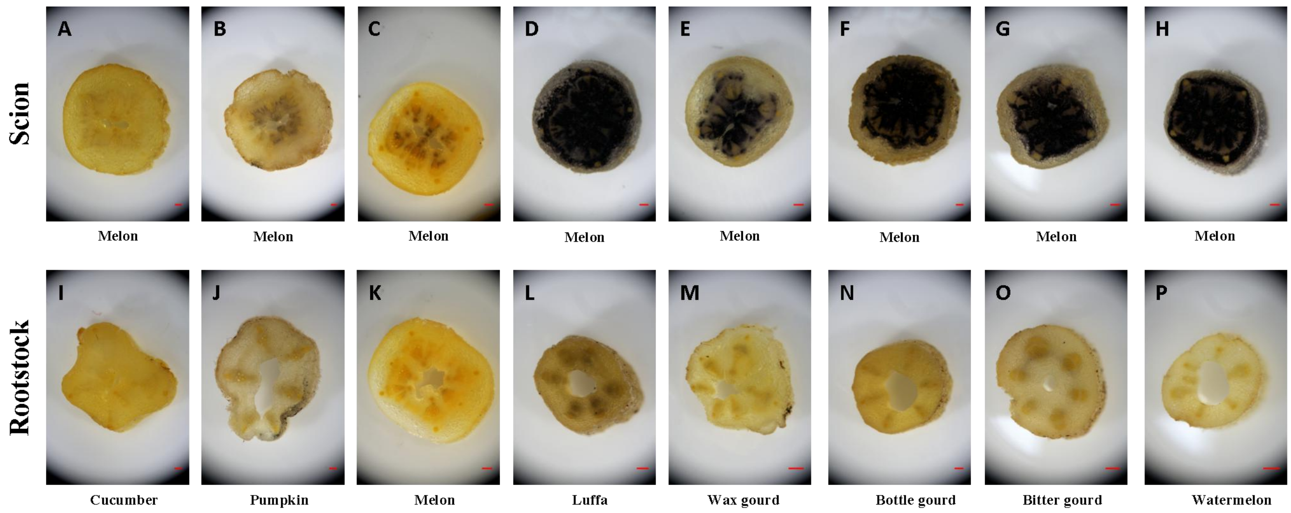

Figure S5. Pictures of starch-iodine above and below the graft junction of melon grafted onto 8 *Cucurbitaceae* species at day 42 after grafting. (A-H) Starch-iodine staining of scion stem above 2 mm the graft junction of melon cv. 'Akekekouqi' grafted onto cucumber cv. 'Jinyou No.35', pumpkin cv. 'Qingyouzhen No.1', melon cv. 'Akekekouqi', luffa cv. 'Sanbi No.6', wax gourd cv. 'Aonong', bottle gourd cv. 'H19', bitter gourd cv. 'Liangku No.1', watermelon cv. 'Zaojia 8424'. Scale bar represents 500 µm. **(I-P)** Starch-iodine staining of rootstock stem below 2 mm the graft junction of melon cv. 'Akekekouqi' grafted onto cucumber cv. 'Jinyou No.35', pumpkin cv. 'Qingyouzhen No.1', melon cv. 'Akekekouqi', luffa cv. 'Sanbi No.6', wax gourd cv. 'Aonong', bottle gourd cv. 'H19', bitter gourd cv. 'Liangku No.1', and watermelon cv. 'Zaojia 8424'. Scale bar represents 500 µm.
